# Efficacy of engineered scaffolds for horizontal and vertical ridge augmentation: A systematic review and meta‐analysis

**DOI:** 10.1002/jper.70103

**Published:** 2026-09-14

**Authors:** Muhammad H. A. Saleh, Elena Calciolari, Ethan Ng, Sandra Stuhr, Hamoun Sabri, Nikolaos Donos, Purnima S. Kumar

**Affiliations:** ^1^ Department of Periodontics and Oral Medicine University of Michigan School of Dentistry Ann Arbor Michigan USA; ^2^ Centre for Oral Clinical Research Institute of Dentistry Faculty of Medicine and Dentistry Queen Mary University of London (QMUL) London UK; ^3^ Department of Medicine and Surgery Centre of Dentistry University of Parma Parma Italy; ^4^ Department of Restorative Dentistry National Dental Centre Singapore Singapore Singapore; ^5^ Center for Clinical Research and Evidence Synthesis in Oral Tissue Regeneration (CRITERION) Ann Arbor Michigan USA

**Keywords:** alveolar ridge augmentation, dental implants, humans, systematic review, tissue scaffolds

## Abstract

**Background:**

Engineered scaffolds hold promise for personalizing surgical therapy and are being increasingly applied in clinical practice. Therefore, a comprehensive synthesis of existing data on ridge augmentation is warranted.

**Methods:**

A systematic review and meta‐analysis were conducted following PRISMA guidelines (PROSPERO: CRD42024611887). Four databases (MEDLINE, EMBASE, Cochrane Central, Scopus) and the grey literature were searched. Eligible studies included randomized clinical trials and prospective case series. The primary outcomes were horizontal, vertical, and volumetric bone gains. A meta‐analysis was performed to obtain pooled estimates for vertical bone gain (mm), horizontal bone gain (mm), volumetric bone gain (mm^3^), and early and late complication rates (%). The certainty of evidence was evaluated using the GRADE approach.

**Results:**

Thirteen studies (*n* = 241 patients, mean age 47.1 years) and 202 defects (12.4% horizontal, 27.2% vertical, and 60.4% combined) were included. Risk of bias was significant, with three of six randomized clinical trials showing low risk and all non‐randomized studies showing moderate‐to‐serious risk of bias. Nine studies used customized computer‐aided design and manufacturing (CAD/CAM) titanium mesh devices, while the remaining studies used materials like polyether ether‐ketone scaffolds (two studies), three‐dimensional hydroxyapatite blocks (one study), or customized allogeneic bone blocks (one study). Pooled horizontal bone gain was 4.96 mm (95% CI: 4.48–5.44 mm), vertical gain 5.64 mm (95% CI: 5.26–6.02 mm), and volumetric gain 1307 mm^3^ (95% CI: 1000–1700 mm^3^) over a 6 to 9‐month follow‐up period. The overall complication rate, irrespective of timing and resorbable membrane use, was 21.7% [16.4–27.0]. The pooled early and late (<4 or ≥4 weeks) complication rates were 24.3% and 16.5%, respectively. Based on six studies, the early complications were 25.6% without versus 21.4% with resorbable membranes. Meta‐regression identified smoking status, surgical experience of the operator, and the defect type as significant factors influencing bone gain.

**Conclusions:**

Engineered scaffolds show promise in managing complex alveolar defects, achieving significant bone gains in all dimensions. However, a high complication rate makes the outcomes unpredictable and limits their use to selective cases.

**Plain language summary:**

This review investigated the usefulness of patient‐specific scaffolds in improving our ability to rebuild lost jawbone. Researchers included 13 studies using different scaffolds, such as custom‐made titanium meshes, polyether ether‐ketone scaffolds, three‐dimensionally printed bone‐like blocks, or customized donated bone blocks. On average, patients gained about 5 mm of new bone in both width and height, and a significant increase in overall bone volume after 6 to 9 months. However, complications were common, affecting about one in five patients. Using a resorbable membrane helped reduce early problems. Key factors such as the surgeon's experience, the type of bone defect (combined horizontal and vertical defects vs. purely vertical), and whether the patient smoked had a strong impact on both bone gain and complications. These findings demonstrated that engineered scaffolds can successfully rebuild missing jawbone, but because complications are relatively frequent, they should be used only in carefully selected patients by highly experienced surgeons.

## 1 INTRODUCTION

Alveolar ridge augmentation (ARA) is a fundamental regenerative technique in implant dentistry, critically addressing vertical and horizontal bone deficiencies that result from tooth extraction, trauma, periodontitis, or pathologic conditions.^1^, ^2^, ^3^ Traditional bone augmentation methods, particularly those involving autogenous bone grafts, have long been favored due to their superior osteogenic potential.^4^, ^5^ Nonetheless, significant drawbacks, such as donor‐site morbidity, prolonged surgical time, postoperative complications, and varying degrees of bone resorption post‐augmentation, pose substantial challenges to clinicians and patients.^6^ Currently, guided bone regeneration (GBR) with different types of barriers and bone grafts/substitutes is the most widely adopted regenerative technique when it comes to ARA.^7^, ^8^

The concept of protected bone healing, primarily through space maintenance, has been crucial in advancing regenerative outcomes in ARA.^9^ Effective bone regeneration requires maintaining sufficient space to allow the migration and differentiation of osteogenic cells and to prevent the collapse of soft tissue into the defect area, as well as the displacement of the bone substitute following flap closure.^10^, ^11^ Traditionally, titanium meshes, reinforced membranes, tenting screws, and bone shells have been employed as rigid space‐maintaining devices.^11^, ^12^ Other forms of titanium frameworks are gaining popularity due to their mechanical stability, ability to be customized, and capability to provide consistent and predictable volume retention.^13^, ^14^ However, while beneficial, the clinical use of these devices is not devoid of complications, including soft tissue dehiscence, membrane exposure, and potential infections, which may jeopardize the regenerative outcome.^15^, ^16^

Advancements in digital technologies (DT), including three‐dimensional (3D) imaging, computer‐aided design and manufacturing (CAD/CAM), and additive manufacturing (3D printing), have transformed the approach to bone augmentation procedures.^17^, ^18^ The advent of digitally designed patient‐specific titanium meshes produced by direct metal laser sintering represents a significant advancement aimed at addressing several customization limitations.^15^ These technologies facilitate precise pre‐surgical planning, allow the fabrication of customized surgical templates and scaffolds, and have thus opened new avenues in GBR.^19^ The development of these customized scaffolds and meshes through CAD/CAM technology has sought to address challenges faced with non‐customized and chair‐side customized scaffolds by enhancing device adaptation and reducing intraoperative adjustments, thereby potentially minimizing complications associated with traditional methods.^20^ They also serve as promising alternatives due to their capability to maintain adequate space for regeneration, enhanced vascularization, and improved clinical handling.^21^ Additionally, the concept of prosthetically guided regeneration (PGR), featuring the significance of integrating prosthetic‐driven digital workflows into regenerative procedures, ensures the optimal placement of implants and restoration of anatomical contours.^17^

Conventional allogeneic bone blocks are typically provided in standardized forms that must be manually shaped to fit the recipient site during surgery.^22^ However, customized allogeneic bone blocks (CABBs), also manufactured via CAD/CAM technology, have been suggested as viable alternatives to autogenous grafts, demonstrating considerable bone gain and reduced donor‐site morbidity.^23^ These tools allow the precise milling of allogeneic bone blanks into customized grafts tailored to the patient's specific defect, thereby eliminating the need for intraoperative manual adaptation.^24^ The clinical efficacy of allogeneic bone blocks has been evaluated in several studies.^25^, ^26^ Various other materials have emerged as appealing alternatives to titanium, either due to their mechanical properties like polyether ether‐ketone (PEEK),^27^, ^28^ or their absorbability, like polylactic acid and hydroxyapatite.^29^, ^30^

Despite encouraging preliminary data, a gap remains in high‐quality clinical evidence comparing these innovative engineered scaffolds to conventional grafting methods and materials.^19^ Given the growing interest in engineering scaffolds and their potential to personalize surgical therapy, a thorough synthesis of existing clinical data is critical. Thus, this systematic review and meta‐analysis aimed to evaluate and compare engineered scaffolds’ clinical efficacy and safety outcomes versus conventional methods in horizontal and vertical alveolar ridge augmentation.

## 2 METHODS

The protocol of this systematic review was registered in the International Prospective Register of Systematic Reviews (PROSPERO) with the identification code CRD42024611887. This review adheres to the guidelines of the Preferred Reporting Items of Systematic Reviews and Meta‐Analyses (PRISMA) 2020 statement.

The following PICOS framework was designed to address four focused questions (FQs):

FQ 1: In patients undergoing horizontal bone augmentation with simultaneous implant placement, what is the horizontal bone gain when using engineered scaffolds (with/without bone grafts/substitutes or membranes) compared to procedures without scaffolds or using other materials (bone grafts/substitutes, membranes, shells, or titanium meshes)?
FQ 2: In patients undergoing horizontal bone augmentation with staged implant placement, what is the horizontal bone gain with engineered scaffolds (with/without bone grafts/substitutes or membranes) compared to procedures without scaffolds or using other materials?
FQ 3: In patients undergoing vertical ridge augmentation with simultaneous implant placement, what is the vertical bone gain with engineered scaffolds (with/without bone grafts/substitutes or membranes) compared to procedures without scaffolds or using other materials?
FQ 4: In patients undergoing vertical ridge augmentation prior to implant placement, what is the vertical bone gain with engineered scaffolds (with/without bone grafts/substitutes or membranes) compared to procedures without scaffolds or using other materials?
**PICOS outline**
  - POPULATION
FQ1–
**Horizontal Alveolar Ridge Augmentation With Simultaneous Implant Placement (HARA‐SI)**
      Systemically healthy, partially dentate adults (aged ≥18 years) with ridge deficiency in the jaws requiring horizontal alveolar bone augmentation to be performed simultaneously with implant placement. For patients with combined horizontal/vertical defects, only the horizontal component is considered for this question.
FQ2 –
**Horizontal Alveolar Ridge Augmentation With Staged Implant Placement (HARA‐ST)**
      Systemically healthy, partially dentate adults (aged ≥18 years) with ridge deficiency in the jaws requiring staged horizontal alveolar bone augmentation. For patients with combined horizontal/vertical defects, only the horizontal component is considered for this question.
FQ3 –
**Vertical Alveolar Ridge Augmentation With Simultaneous Implant Placement (VARA‐SI)**
      Systemically healthy, partially dentate adults (aged ≥18 years) with ridge deficiency in the jaws requiring vertical alveolar bone augmentation to be performed simultaneously with implant placement. For patients with combined horizontal/vertical defects, only the vertical component is considered for this question.
FQ4 –
**Vertical Alveolar Ridge Augmentation With Staged Implant Placement (VARA‐ST)**
      Systemically healthy, partially dentate adults (aged ≥ 18 years) with ridge deficiency in the jaws requiring staged vertical alveolar bone augmentation. For patients with combined horizontal/vertical defects, only the vertical component is considered for this question.
  - INTERVENTION:
    ARA with an engineered scaffold. For this manuscript, an ‘engineered scaffold’ was defined as having the following characteristics:
    - Custom‐designed using CAD/CAM technology to match patient‐specific alveolar bone defects.
    - Can be fabricated as blocks or barriers (e.g., PEEK, titanium mesh, polylactic acid)
    - May be used alone or in combination with any bone replacement graft (autologous, allogenic, xenogeneic, or synthetic) with or without a barrier membrane.
    - May be enhanced with osteoinductive factors (e.g., bone morphogenetic proteins^31^).
    Regenerative surgeries with cell therapy were excluded.

- COMPARISON:
  The same surgical procedure without the use of an engineered scaffold.

- OUTCOMES:
**HARA‐SI and VARA‐SI**
    Primary Outcome: Defect resolution (%) at re‐entry or at implant placement, measured by clinical or radiographic (CBCT) assessment.
**HARA‐ST and VARA‐ST**
    Primary Outcome: Bone gain (horizontal, vertical, and/or volumetric) measured clinically or radiographically (CBCT) at re‐entry.
**All Groups**
    Secondary outcomes: Additional metrics, including complication rates (e.g., exposure of the engineered scaffold, infection) reported as early (< 4 weeks) or late (≥ 4 weeks),^18^ patient‐reported outcomes (PROMs),^32^ bone quality, and density.^33^
- STUDY DESIGN:
  Any randomized clinical trial (RCT), prospective clinical study (CCT), or prospective case series with ≥4‐month follow‐up post‐regenerative procedure and a minimum of eight patients.

### 2.1 Information sources and search strategy

A systematic search strategy was developed to identify all studies that met the inclusion/exclusion criteria. The search strategy included terms related to the Population and the Intervention/ Comparison investigated in this review, combined with the Boolean operators “AND” and “OR” (Table S1). Three main databases and one data registry were searched: The National Library of Medicine (MEDLINE via PubMed). EMBASE via OVID, Scopus (Elsevier), and the Cochrane Central Register of Controlled Trials. Bibliographies of review articles on this topic and of all studies included for data extraction were screened and the database Web of Science was used to identify all the papers that cited the included papers. To search for unpublished trials, the grey literature, nonprofit reports, government research, or other materials were also electronically explored, using ClinicalTrial.gov and OpenGrey (www.opengrey.eu).

No restrictions were applied regarding the publication date, journal, or language used. In the attempt to include both published and unpublished data, a specific theses database, www.theses.com/ was searched and an additional hand search was performed for the last 2 years for the journals that published more about this topic (*Journal of Dental Research*, *Journal of Clinical Periodontology*, *Journal of Periodontology*, *Clinical Oral Implants Research*, *Clinical Implant Dentistry and Related Research*, *Journal of Periodontal Research*, *Clinical Oral Investigations*, *International Journal of Oral and Maxillofacial Implants*, and *International Journal of Periodontics and Restorative Dentistry*).

### 2.2 Selection process

All records were imported into EndNote (version 21, Clarivate, Philadelphia, PA) to remove duplicates and then transferred to Rayyan software for screening (Rayyan, Qatar Foundation, Qatar).^34^ A two‐stage selection process was carried out independently and in duplicate by two reviewers (M.H.S. and E.N.), who were not blinded to the author or institutions. First, studies were assessed based on their titles and abstracts, and those meeting the inclusion criteria were then screened for full‐text analysis. After completing the screening process, both reviewers individually went through the full‐text version of the potentially eligible studies, and final article selection was dictated by the eligibility criteria. Inter‐examiner calibration was achieved by open discussion and comparison after independent assessment of the first 300 records. In the event of disagreements, both reviewers had an open discussion, failing which, another co‐author (P.K. and/or N.D.) made the final decision. For studies on the same patient population, only the article reporting the longest follow‐up was selected for quantitative analysis.

### 2.3 Data extraction

Data were extracted independently by four examiners (M.H.S., E.C., E.N., and S.H.S.) using a standardized data extraction form. Prior to data extraction, the examiners were calibrated by using a random selection of five articles to ensure consistency in the data extraction process and the terminology used. Final data accuracy and consistency were independently verified by an additional author (H.S.). Where applicable, any missing information that could contribute to this review was requested by the corresponding author(s) via email communication.

The primary unit of analysis was the defect/site. When a study reported multiple sites per patient, we extracted site‐level outcomes where available. A given defect contributed only once to a given meta‐analysis. For combined defects, horizontal measurements were included only in horizontal analyses and vertical measurements only in vertical analyses to avoid any double counting.

### 2.4 Methodological quality and risk‐of‐bias assessment

The assessment of methodological quality and risk of bias of each included RCT was performed by two independent reviewers (E.C. and S.H.S.) using version 2 of the Cochrane risk‐of‐bias tool for RCTs (RoB2)^35^ and the ROBINS‐I V2 tool^36^ for non‐randomized studies.

For non‐randomized controlled trials, the assessment of methodological quality and risk of bias was performed by the same independent reviewers (E.C. and S.H.S.) using version 2 of the Risk of Bias in Non‐Randomized Studies of Interventions (ROBINS‐I V2).^35^ ROBINS‐I V2 assesses the following types of bias in seven domains: (1) risk of bias due to confounding; (2) risk of bias in classification of intervention; (3) risk of bias in selection of participants into the study (or into the analysis); (4) risk of bias due to deviations from intended interventions; (5) risk of bias due to missing data; (6) risk of bias arising from measurement of the outcome; and (7) risk of bias in selection of the reported result.

### 2.5 Data synthesis

Extracted data were organized into evidence tables. In addition to the reported outcomes of interest, supplemental data included year of publication and author(s), country(ies) and setting(s) in which the study was conducted, ethical approval reporting, study design, initial and final number of participants, sex and age distribution, and description of intervention(s). Other outcomes of interest included surgical complications and timing of complications, patient‐reported outcome measures (PROMs), histological parameters, and implant‐related outcomes relevant to this review.

### 2.6 GRADE assessment of certainty of evidence

The certainty (quality) of evidence was evaluated using the Grading of Recommendations Assessment, Development and Evaluation (GRADE) framework, which provides a systematic and transparent approach for rating the quality of evidence across outcomes.^37^ According to GRADE principles, the certainty of evidence for each outcome was assessed by considering the number of studies, study design, and risk of bias using RoB 2.0 and ROBINS‐I V2 tools. Inconsistency was evaluated by examining variability in outcome estimates across studies and subgroups. Indirectness was assessed based on the applicability of the populations, interventions, and outcomes to the review question. Imprecision was judged based on the width of the confidence intervals and sample sizes. Publication bias was not formally assessed due to the nature of the pooled data, which did not involve treatment effect estimates. The overall certainty of evidence was graded as high, moderate, low, or very low, depending on the presence and magnitude of concerns across these domains.

### 2.7 Statistical analysis

A meta‐analysis was performed to obtain pooled estimates for vertical bone gain (mm), horizontal bone gain (mm), volumetric bone gain (mm^3^), and early and late complication rates (%). A random‐effects model using the restricted maximum likelihood (REML) estimator was applied to account for between‐study heterogeneity. Inverse‐variance weighting was used for pooling effect sizes across studies. For continuous outcomes, the weighted mean and standard deviation were pooled, whereas for early complication rates, a proportional meta‐analysis was performed. Between‐study heterogeneity was assessed using Cochran's Q‐test (*p* < 0.10 considered significant) and the *I* ^2^ statistic (low: < 25%, moderate: 25%–50%, high: >50%). Subgroup analyses were conducted based on resorbable membrane use (Yes vs. No) and scaffold material type (CAD‐CAM titanium mesh, PTFE, PEEK shells).

A meta‐regression analysis was then performed, adjusting for study design (RCT vs. case series), defect type (horizontal vs. combined), augmentation material, resorbable membrane use, follow‐up time, patient‐related factors (age, smoking status, and sex distribution), and clinician experience level (graduate students vs. expert surgeons) on the outcome of bone gain. A mixed‐effects meta‐regression model was applied, incorporating random effects at the study level (Study ID as a random intercept) to account for within‐study correlations.

Forest plots were generated to visualize pooled estimates for bone gain outcomes and early complication rates, ensuring clear separation of subgroups and adjusting for column spacing to improve readability. Publication bias was assessed using Egger's test.

All statistical analyses were performed using RStudio software (Version: 2024.12.1+563) with the meta, metafor, and forestploter packages. A significance threshold of *p* < 0.05 was applied, and all *p*‐values from meta‐regression models were adjusted for multiple comparisons using the Benjamini–Hochberg false discovery rate (FDR) correction. Statistical analyses were conducted by one author (H.S.).

## 3 RESULTS

### 3.1 Study selection

A total of 16,539 records were identified from the systematic search of four databases, and the grey literature. Of the 10,509 records screened after the removal of duplicates, 13 studies^18^, ^27^, ^28^, ^38^, ^39^, ^40^, ^41^, ^42^, ^43^, ^44^, ^45^, ^46^, ^47^ and 15 articles^18^, ^27^, ^28^, ^38^, ^39^, ^40^, ^41^, ^42^, ^43^, ^44^, ^45^, ^46^, ^47^, ^48^, ^49^ were included in the review, as two studies^48^, ^49^ were additional analyses of an original study^18^ (The PRISMA flow chart can be found in Figure S1). Finally, nine studies were included for quantitative analysis.^18^, ^27^, ^38^, ^39^, ^41^, ^42^, ^44^, ^45^, ^47^ Among the 13 included studies, none answered Focused Questions 1 or 3. Three studies exclusively answered Focused Question 2,^39^, ^42^, ^47^ three studies exclusively answered Focused Question 4,^40^, ^43^, ^46^ and seven studies answered both Focused Questions 2 and 4.^18^, ^27^, ^28^, ^38^, ^41^, ^44^, ^45^

Table S2 presents a table that demonstrates the excluded articles after full‐text assessment, along with the reasons for exclusion. Table S3 shows the included studies after full‐text assessment in both qualitative and quantitative assessments.

### 3.2 Study characteristics

Thirteen studies were included in the qualitative analysis^18^, ^27^, ^28^, ^38^, ^39^, ^40^, ^41^, ^42^, ^43^, ^44^, ^45^, ^46^, ^47^ (see Supporting Information). The study designs of the included studies were as follows: seven prospective case series^27^, ^38^, ^39^, ^40^, ^41^, ^42^, ^43^ and six single‐center RCTs.^18^, ^28^, ^44^, ^45^, ^46^, ^47^ Seven studies were performed in Italy,^18^, ^38^, ^40^, ^41^, ^43^, ^44^, ^46^ three in Egypt,^27^, ^28^, ^39^ one in Thailand,^42^ one in Bulgaria,^45^ and one in China.^47^ With regard to the study setting, three studies^38^, ^40^, ^41^ were conducted in hospitals, and the remaining 10 studies^18^, ^27^, ^28^, ^39^, ^42^, ^43^, ^44^, ^45^, ^46^, ^47^ took place in universities.

The total number of patients enrolled from all included studies was 241. The weighted mean age reported across 11 studies^18^, ^28^, ^38^, ^39^, ^40^, ^41^, ^42^, ^43^, ^44^, ^45^, ^46^, ^47^ (*n* = 171 patients) was 47.1 years (ranging from 28.3 to 56.37). None of the studies provided data on bruxism, parafunctional habits, or traumatic occlusal forces. Smoking status was reported by five studies,^38^, ^43^, ^44^, ^46^, ^47^ and no heavy smokers were included. Periodontal status was reported by three studies,^42^, ^46^, ^47^ and was controlled before enrollment in all studies.

All studies used a staged regeneration protocol. The weighted analysis of defect distribution (among studies that provided quantitative data) revealed that among 178 defects, 91 (51.1%) were in the maxilla and 87 (48.9%) in the mandible. Out of the 202 total defects, pure horizontal defects were (12.4%); pure vertical defects were (27.2%); and combined (horizontal and vertical), summed up to 122 defects (60.4%).

In some studies,^45^, ^47^ details were not provided and were therefore excluded from the weighting calculations.

Surgeon's experience was reported in 10 of the included studies. Six studies stated that all surgical procedures were performed by an expert surgeon(s).^18^, ^28^, ^43^, ^44^, ^46^, ^47^ Lizio et al. (2022) reported that surgeons with different expertise and experience performed procedures,^38^ while three studies described surgery being performed by postgraduate students under close supervision.^27^, ^39^, ^42^

### 3.3 Intervention characteristics

Nine of the 13 included studies^18^, ^38^, ^39^, ^40^, ^41^, ^43^, ^44^, ^45^, ^46^ used titanium‐based scaffolds/devices (e.g., CAD/CAM customized titanium mesh). The other four studies used variable scaffold constructs like CAD/CAM in‐house printed PEEK scaffolds,^27^, ^28^ 3D hydroxyapatite blocks,^42^ or a customized allogenic bone block (CABB).^47^ Within these groups, a mixture of autogenous and xenogeneic bone was used in 11 studies (84.6%)^18^, ^27^, ^28^, ^38^, ^39^, ^40^, ^41^, ^43^, ^44^, ^45^, ^46^ with different ratios. The remaining two studies used only particulate bovine bone^42^ or allograft bone particulate.^47^

### 3.4 Meta‐analysis on the primary outcome of bone gain

#### 3.4.1 Horizontal bone gain (three studies)

**FIGURE 1:**
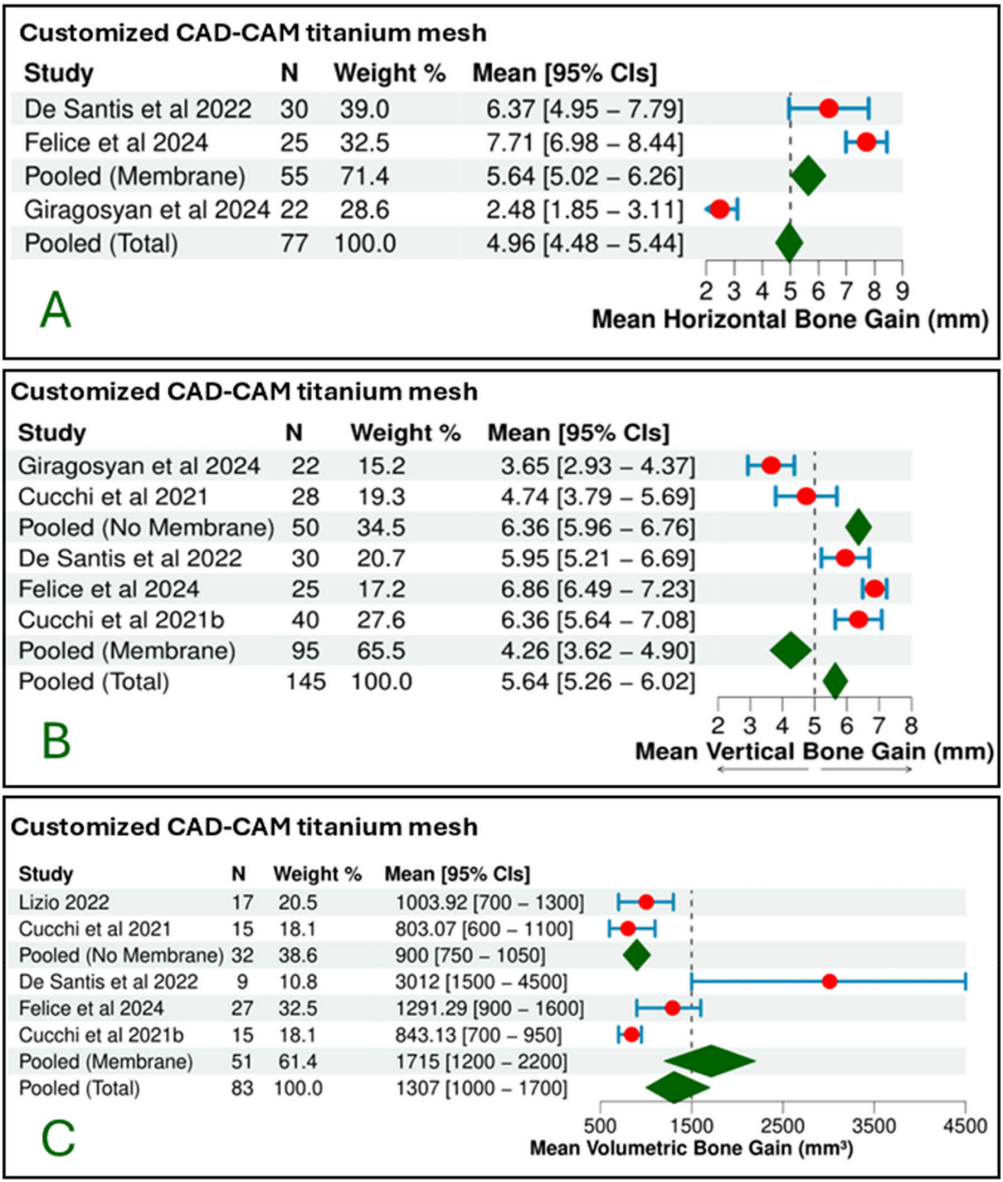
Forest plots showing pooled mean horizontal (A), vertical (B), and volumetric (C) bone gain (mm/mm^3^) across studies reporting this outcome.

The pooled analysis of horizontal bone gain demonstrated a total mean of 4.96 mm (95% CI: 4.48–5.44) following a 4‐ to 10‐month healing period. Subgroup analysis revealed that the use of customized CAD‐CAM titanium mesh with a resorbable membrane (two studies, 55 cases)^41^, ^44^ resulted in a greater mean horizontal bone gain of 5.64 mm (95% CI: 5.02 – 6.26), whereas cases without a resorbable membrane (1 study, 22 cases)^44^ showed a significantly lower gain of 2.48 mm (95% CI: 1.85–3.11). The highest reported gain was observed in the study by Felice et al. (2024) utilizing a resorbable membrane, with a mean of 7.71 mm (95% CI: 6.98–8.44) after an 8‐month follow‐up period (Figure 1A).

#### 3.4.2 Vertical bone gain (five studies)

The pooled analysis of vertical bone gain across 145 cases showed a mean of 5.64 mm (95% CI: 5.26–6.02). Subgroup analysis indicated that cases without a resorbable membrane (two studies, 50 cases)^18^, ^44^ exhibited a higher mean gain of 6.36 mm (95% CI: 5.96–6.76), whereas cases with a resorbable membrane (three studies, 95 cases)^18^, ^41^, ^44^ demonstrated a lower mean gain of 4.26 mm (95% CI: 3.62–4.90). The highest vertical gain reported in a study with a resorbable membrane was 6.86 mm (95% CI: 6.49–7.23) (Figure 1B).

#### 3.4.3 Volumetric bone gain (five studies)

The pooled analysis of volumetric bone gain when customized CAD/CAM titanium mesh was used across 83 cases revealed a mean of 1307 mm^3^ (95% CI: 1000–1700). Subgroup analysis indicated that cases without a resorbable membrane (two studies, 32 cases)^18^ had a lower mean volume gain of 900 mm^3^ (95% CI: 750–1050), while those with a resorbable membrane (three studies, 51 cases)^18^, ^41^, ^44^ demonstrated a higher mean gain of 1715 mm^3^ (95% CI: 1200–2200). The highest individual mean volume gain was reported at 3012 mm^3^ (95% CI: 1500–4500) (Figure 1C).

### 3.5 Secondary outcomes

#### 3.5.1 Early complications

**FIGURE 2:**
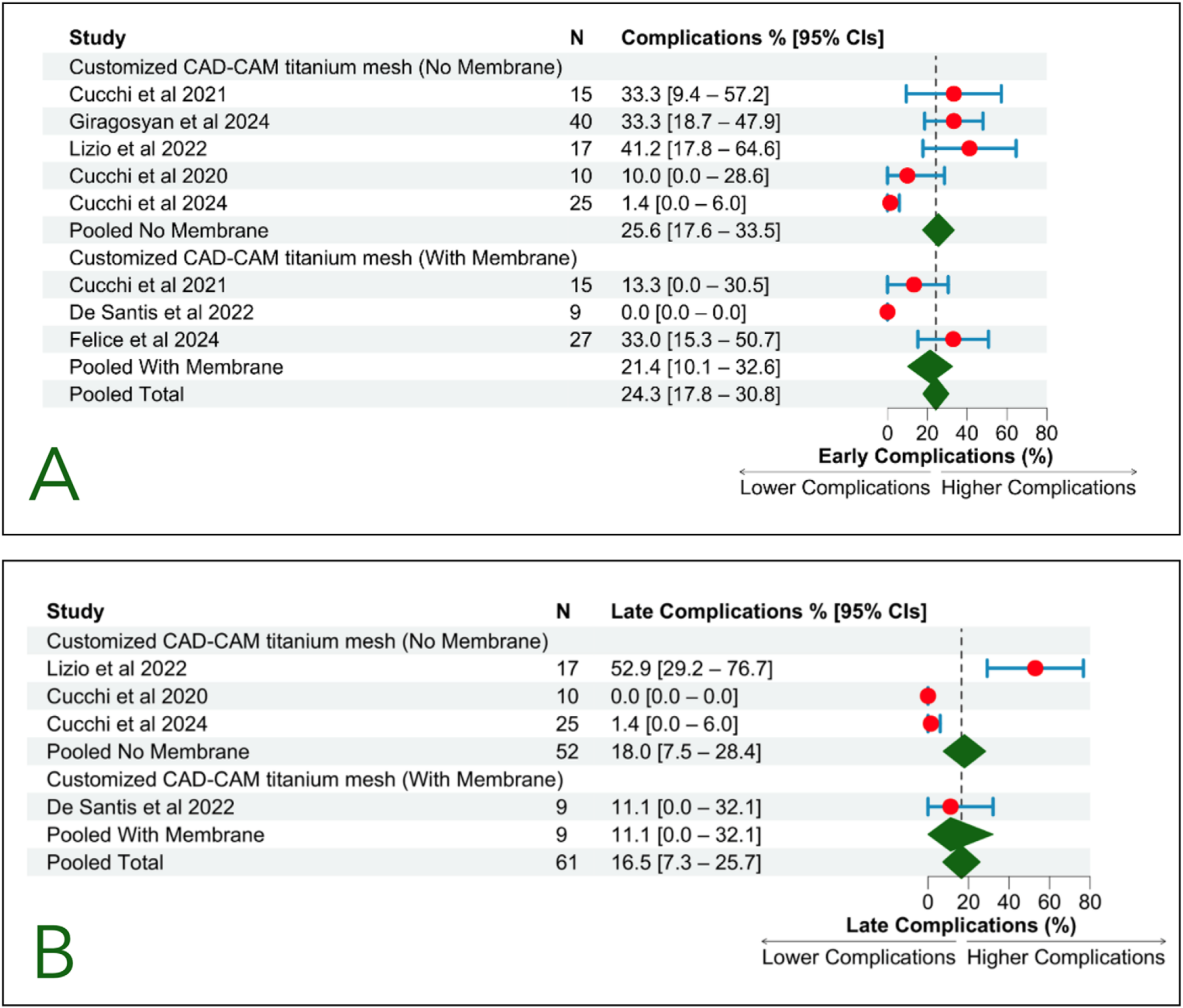
Forest plots showing pooled early (A) and late (B) complication rates (%). Early: no membrane 25.6% (95% CI: 17.6–33.5) versus membrane 21.4% (10.1–32.6); difference not significant. Late: no membrane 18.0% (7.5–28.4) versus membrane 11.1% (0.0–32.1); difference not significant.

The overall complication rate, irrespective of timing and resorbable membrane use, was 21.7% [16.4–27.0]. The pooled analysis of early complication rates across 123 cases using customized CAD‐CAM titanium mesh demonstrated an overall early complication rate of 24.3% (95% CI: 17.8%–30.8%). Subgroup analysis showed that cases without a resorbable membrane (72 cases) had a higher complication rate of 25.6% (95% CI: 17.6%–33.5%), whereas cases incorporating a resorbable membrane (51 cases) exhibited a lower rate of 21.4% (95% CI: 10.1%–32.6%). Individual study‐level data showed variations, with the highest complication rate reported at 41.2% (95% CI: 17.8%–64.6%),^38^ while one study observed no early complications^41^ (Figure 2A).

#### 3.5.2 Late complications

Within the CAD‐CAM titanium mesh subgroup (113 cases), the pooled overall late complication rate was 16.5% (95% CI:7.3%–25.7%), with notable variability between individual studies. Lizio et al. reported the highest late complication rate at 52.9% (95% CI: 29.2%–76.7%),^38^ (CAD‐CAM titanium mesh without a resorbable membrane), while De Santis et al. (CAD‐CAM titanium mesh with a resorbable membrane) observed a markedly lower rate of 11.1% (95% CI: −9.4% to 31.6%).^41^ Meanwhile, El‐Morsy et al. (CAD‐CAM PEEK shells) reported a 7.0% late complication rate (95% CI: −6.4% to 20.4%)^27^ (Figure 2B).

#### 3.5.3 Patient‐reported outcomes (PROMs)

Only two studies reported on PROMs, which indicated generally low/moderate patient‐reported pain (VAS score or numerical rating scale) during surgery time and over the follow‐up periods, regardless of the surgical technique employed for horizontal ridge augmentation.^42^, ^47^

#### 3.5.4 Histological analysis, bone quality, and density

Three studies reported data related to bone quality/density. In particular, when performing vertical augmentation with a mix of autologous and xenogeneic bone combined with a CAD/CAM customized titanium mesh or with a Ti‐reinforced d‐PTFE mesh, the majority of the regenerated sites presented medium or hard density.^18^, ^43^, ^48^

Three studies reported histomorphometric data^39^, ^42^, ^48^ with a heterogeneous amount of regenerated bone. In particular, in cases requiring horizontal ridge augmentation, a percentage of regenerated bone ranging from 28.6% ± 1.88%^42^ to 79.6% ± 29.2%^39^ at 4–6 months was reported, while Cucchi et al. reported a mean 34.82% ± 10.13% bone formation at 6 months in combined horizontal and vertical regeneration cases.^48^ Moreover, one study qualitatively assessed the regenerated bone and indicated similar histological features when customized allogeneic bone blocks were used for alveolar ridge augmentation compared to autogenous bone blocks.^47^ Only one study utilized micro‐CT to evaluate bone morphology and structure with CAD/CAM‐customized titanium mesh^18^, ^49^ (Table S4).

### 3.6 Meta‐regression

#### 3.6.1 Horizontal bone gain

In the analysis of horizontal bone gain, none of the examined variables demonstrated a statistically significant effect, except for smoking status and the surgeon's experience level. Light smokers exhibited significantly less horizontal bone gain compared to non‐smokers, with a mean difference of −2.4 mm (95% CIs: −4.23 to −1.26, *p* = 0.031). Analysis at the level of surgical experience (graduate students as the reference group) indicated 2.42 mm more bone gain when expert surgeons performed the surgery (estimate: 2.42 mm; 95% CIs: 0.91–5.48, *p* = 0.021) (Table 1). Other factors, including arch location, surgical time, defect type, graft material, study design, age, and sex, did not show significant associations with horizontal bone gain (*p* > 0.05).

**TABLE 1:** Mixed‐effects meta‐regression model results on outcome of horizontal bone gain (mm).

	Univariate meta‐regression analysis			
Variable	Estimate	SE	95% CIs [Lower—Upper]	*p* value
Maxilla (%)	−0.0055	0.0351	−0.0743 to 0.0633	0.8755
Time (months)	0.0393	0.1731	−0.2999 to 0.3785	0.8202
Defect type (horizontal vs. combined)	−1.2081	1.5741	−4.2933 to 1.8771	0.4428
Group				
Customized CAD‐CAM titanium mesh	0.84	3.2938	−5.6158 to 7.2957	0.7987
Customized CAD‐CAM titanium mesh + RM	2.7353	3.3376	−3.8063 to 9.2768	0.4125
Non‐perforated CAD/CAM Ti‐Mesh	0.6459	3.474	−6.163 to 7.4549	0.8525
Non‐perforated CAD/CAM Ti‐Mesh + RM	2.6236	3.424	−4.0873 to 9.3345	0.4435
RM	0.3312	3.6146	−6.7532 to 7.4156	0.927
RM + PRF	−0.9	3.4751	−7.7111 to 5.9111	0.7956
ti‐PTFE	1.1863	3.1497	−4.987 to 7.3596	0.7064
Graft material				
Autogenous block + Allograft	−0.03	3.6192	−7.1235 to 7.0635	0.9934
Autogenous particles + Xenograft	2.0442	2.1675	−2.204 to 6.2924	0.3456
CABB + Allograft	1.584	2.98	−4.2567 to 7.4247	0.595
Study design (RCT vs. CS)	0.7928	1.522	−2.1901 to 3.7758	0.6024
Age	0.0483	0.0668	−0.0826 to 0.1792	0.4694
Sex (Male %)	0.0011	0.0343	−0.0662 to 0.0683	0.9748
Smoking (light smokers vs. non‐smokers)	−2.429	−4.234	−4.232 to −1.2628	0.031
Surgeon experience (reference: graduate students)				
Expert surgeons	2.422	0.899	0.911 to 5.487	**0.021**

*Note*: Multivariate model was not built because of the limited number of studies and risk of model overfitting.

Abbreviations: CABB, customized allogenic bone block; CI, confidence interval; CS, case series; PRF, platelet‐rich fibrin; RCT, randomized controlled clinical trial; RM, resorbable membrane; SE, standard error.

#### 3.6.2 Vertical bone gain

In the univariate analysis for vertical bone gain, defect type (combined vs. horizontal) was associated with an estimate of 4.42 mm (95% CI: 2.55–6.30, *p* < 0.0001). Surgical time was associated with an estimate of 1.33 mm (95% CI: 0.09–2.58, *p* = 0.0351). Age was estimated at 0.1682 mm (95% CI: 0.05–0.28, *p* = 0.0031). Smoking (light smokers vs. non‐smokers) was associated with an estimate of −1.12 mm (95% CI: −2.73 to −0.21, *p* = 0.001) (Table 2).

**TABLE 2:** Uni‐ and multivariate meta‐regression analysis on the outcome of vertical bone gain (mm).

	Univariate meta‐regression analysis				Multivariate meta‐regression analysis			
Variable	Estimate	SE	95% CIs [Lower—Upper]	*p* value	Estimate	SE	95% CIs [Lower—Upper]	*p* value
Study design (RCT vs. CS)	−0.169	2.3048	−4.6863 to 4.3483	0.9415				
Defect type								
Combined versus horizontal	4.4292	0.9545	2.5584 to 6.3000	<.0001	3.11	0.99	2.121 to 3.899	0.001
Group								
Customized CAD‐CAM titanium mesh	0.5217	2.046	−3.4884 to 4.5319	0.7987				
Customized CAD‐CAM titanium mesh + RM	3.1918	1.6658	−0.073 to 6.4567	0.0553				
Non‐perforated CAD/CAM Ti‐Mesh + RM	3.34	2.5823	−1.7213 to 8.4013	0.1959				
RM	−2.4382	1.8762	−6.1155 to 1.239	0.1937				
ti‐PTFE	2.831	1.6159	−0.3362 to 5.9982	0.0798				
Graft material (Ref. CABB + Allograft)								
Autogenous block + Allograft	−0.84	2.3918	−5.5278 to 3.8478	0.7254				
Autogenous particles + Xenograft	3.9628	2.0528	−0.0606 to 7.9862	0.0536				
Time	1.3363	0.6343	0.0931 to 2.5795	0.0351	1.01	0.89	−0.11 to 1.78	0.123
Maxilla (%)	0.044	0.0586	−0.0709 to 0.159	0.4526				
Age	0.1682	0.0569	0.0568 to 0.2797	0.0031	0.09	0.089	−0.12 to 0.11	0.09
Sex (Male %)	0.0235	0.1078	−0.1879 to 0.2348	0.8278				
Smoking (Light vs. non‐smokers)	−1.122	0.891	−2.7376 to −0.217	0.001	−0.781	0.9	−2.11 to −0.111	0.01

Abbreviations: CABB, customized allogenic bone blocks; CI, confidence interval; CS, case series; RCT, randomized controlled clinical trial; RM, resorbable membrane; SE, standard error.

In the multivariate analysis, defect type (combined vs. horizontal) remained significant, with an estimate of 3.11 mm (95% CI: 2.12–3.9, *p* = 0.001). Smoking (light smokers vs. non‐smokers) remained significant, with an estimate of −0.78 mm (95% CI: −2.11 to −0.11, *p* = 0.01). Time and age were not significant in the multivariate analysis (Table 2).

#### 3.6.3 Volumetric bone gain

In the univariate analysis of volumetric bone gain, defect type (horizontal vs. combined) was associated with an estimate of −852.84 (95% CI: −1254.85 to −450.84, *p* < 0.0001). Autogenous particles + xenograft demonstrated a significant positive association with higher volumetric bone gain versus reference group of 3DHA+Xenograft (estimate = 868.0872, 95% CI: 434.75–1301.41, *p* < 0.0001). Defects in maxilla were significantly associated with lower bone gain with an estimate of −26.02 (95% CI: −38.12 to −13.91, *p* < 0.0001). Other variables, including study design, different group materials, time, age, sex, and smoking, did not show statistical significance (*p* > 0.05) (Table 3).

**TABLE 3:** Uni‐ and multivariate meta‐regression analysis on the outcome of volumetric bone gain (mm^3^).

	Univariate meta‐regression analysis				Multivariate meta‐regression analysis			
Variable	Estimate	SE	95% CIs [Lower—Upper]	*p* value	Estimate	SE	95% CIs [Lower—Upper]	*p* value
Study design (RCT vs. CS)	560.8714	432.6023	−287.0135 to 1408.7563	0.1948				
Defect type (combined vs. horizontal)	852.847	205.1083	450.8426 to 1254.8523	<0.0001	720.135	210.4567	286.0307−1154.2383	0.0021
Group (Ref. ti‐PTFE)								
Customized CAD‐CAM titanium mesh	−254.93	562.4591	−1357.3292 to 847.47	0.6504	−189.42	580.32	−1320.45 to 941.61	0.7281
Customized CAD‐CAM titanium mesh + RM	−64.3421	511.4295	−1066.7256 to 938.0414	0.8999	−92.17	495.89	−1058.33 to 874.00	0.8425
Non‐perforated CAD/CAM Ti‐Mesh + RM	177.83	942.9177	−1670.2547 to 2025.9147	0.8504	210.56	910.22	−1543.12 to 1964.24	0.7632
RM + PRF	−945.83	442.9187	−1813.9347 to −77.7253	0.0327	−850.237	410.2389	−1643.3471 to −57.1277	0.0253
Graft Material (Ref: 3DHA + Xenograft)								
Autogenous block + Xenograft	774.12	473.1203	−153.1788 to 1701.4188	0.1018	320.45	510.78	−679.23 to 1320.13	0.327
Autogenous Particles + Xenograft	868.0872	221.0903	434.7582 to 1301.4162	<.0001	745.2934	225.8734	403.2456 to 1087.3412	<.0001
Time	401.9784	240.265	−68.9324 to 872.8891	0.0943				
Maxilla (%)	−26.0233	6.1753	−38.1266 to −13.9199	<0.0001	−21.5784	5.6321	−32.7284 to −10.4283	0.0013
Age	46.3162	105.3726	−160.2104 to 252.8428	0.6603				
Sex (Male %)	−21.9251	27.1072	−75.0543 to 31.204	0.4186				
Smoking (Light vs. non‐smokers)	703.9143	399.5988	−79.2849 to 1487.1134	0.0781				

Abbreviations: 3DHA, 3D‐printed nano‐hydroxy‐apatite; CI, confidence interval; CS, case series; RCT, randomized controlled clinical trial; RM, resorbable membrane; SE, standard error.

In the multivariate analysis, defect type (combined vs. horizontal) remained significant with an estimate of 720.1345 (95% CI: 286.03–1154.23, *p* = 0.0021). RM + PRF remained significant, with an estimate of −850.23 (95% CI: −1643.34 to −57.12, *p* = 0.0253). Autogenous particles + xenograft remained significantly associated with greater volumetric bone gain, with an estimate of 745.29 (95% CI: 403.24–1087.34, *p* < 0.0001). Arch location also remained significant, with an estimate of ‐21.57 (95% CI: −32.72 to −10.42, *p* = 0.0013) (Table 3).

### 3.7 Risk‐of‐bias assessment

Only three of the six randomized controlled trials, with two containing the same study population, exhibited high methodological quality according to the Cochrane RoB 2.0.^35^ Of the remaining three studies, two presented with some concerns^44^, ^46^ and one with a high risk of bias^45^ (Table S5).

None of the non‐randomized studies exhibited a low risk of bias according to the ROBINS‐I V2. Three studies demonstrated a moderate risk of bias,^39^, ^41^, ^43^ while the other four (with two involving the same study population) were considered to be at a serious risk of bias^27^, ^38^, ^40^, ^42^ (Table S6).

### 3.8 Certainty (quality) of evidence

According to the GRADE assessment, the overall certainty of the evidence was rated as moderate for horizontal and vertical bone gain, reflecting some concerns about the risk of bias and moderate imprecision. The certainty was rated as low for volumetric bone gain, early complications, and late complications due to serious concerns regarding inconsistency, imprecision, and risk of bias across contributing studies. Publication bias could not be formally assessed and was rated unclear for all outcomes (Table S7).

## 4 DISCUSSION

This systematic review and meta‐analysis of ARA using customized CAD/CAM techniques revealed that digital planning and milling/3D printing procedures could lead to significant horizontal and vertical bone gain, albeit with a high rate of complications, which compromises the predictability of the procedure. Overall, the analyses demonstrated increased and clinically meaningful horizontal (4.96 mm; 95% CI: 4.48–5.44), vertical (5.64 mm; 95% CI: 5.26–6.02), and volumetric mean bone gains (1307 mm^3^; 95% CI: 1000–1700); (6 to 9‐month follow‐up). The addition of resorbable membranes on top of the titanium mesh appeared to enhance the mean volumetric bone gain and reduce, up to a certain extent, complication rates. Remarkably, patient factors, particularly smoking status and defect type (with combined defects having more vertical bone gains compared with pure vertical defects), played a role in modulating these outcomes. Smoking status (even light smokers), surgical experience, surgical time, and the addition of autograft significantly affected the results of the procedure.

The pooled data indicated a substantial horizontal bone gain that varied slightly according to the use of resorbable membranes over a 6 to 9‐month follow‐up period. Our subgroup analysis based on two studies (32 cases) showed a horizontal gain of 5.64 mm with resorbable membranes, which is somewhat higher than previous studies reporting gains between 3 and 6 mm when using conventional techniques (4.18 ± 0.56 mm for the block graft technique and 3.61 ± 0.27 mm for GBR).^50^ Furthermore, and within the limitation of the low number of studies included in this analysis, the present meta‐regression demonstrated that even light smoking was associated with significantly reduced horizontal bone gain.

Smoking has been shown to adversely affect bone grafting procedures, primarily due to its association with increased resorption rates.^51^, ^52^ This may be due to a direct effect of smoking on wound healing of both bone and soft tissues.^53^, ^54^ Evidence suggests that tobacco may inhibit the proliferation and osteogenic differentiation of marrow‐derived mesenchymal stem cells in the human alveolar bone. As a result, smoking may hinder osseointegration and promote bone resorption around dental implants.^55^ However, not all the studies included attempted to establish a correlation between complications and smoking. This means that the lower bone gain may be due to a direct effect, as mentioned earlier, or as a result of an indirect effect of smoking, which causes other complications such as increased wound dehiscence^56^ or membrane exposure.^57^

Several other surgical factors are critical in determining the success and predictability of regenerative outcomes.^8^ Surgical success depends highly on primary intention and tension‐free wound healing; otherwise, scar tissue formation, volume loss, and compromised esthetic and functional outcomes may occur.^58^ Sutures must be able to withstand mechanical forces during the early healing phases without imposing excessive tension, which could impair microcirculation and compromise wound stability. To this end, the adoption of microsurgical techniques, supported by optical magnification and delicate instrumentation, has been shown to significantly reduce postoperative morbidity while enhancing surgical precision and minimizing tissue trauma.^59^, ^60^

Thick tissue phenotype, along with adequate flap advancement, has been shown to improve GBR outcomes by enhancing graft containment, minimizing the risk of wound opening, facilitating flap mobilization, and enabling tension‐free closure, which helps to mitigate postoperative swelling and decrease the likelihood of membrane exposure.^61^, ^62^ Prolonged surgical manipulation may exacerbate tissue trauma and inflammatory responses, potentially compromising flap stability, particularly when performed by operators with varying levels of experience, pointing to the correlation of more surgical experience with positive surgical outcomes.^63^ In a study evaluating patient‐reported outcomes following GBR, a mean surgical time of approximately 2 h was observed when periodontal residents performed procedures under supervision, and longer surgery duration was significantly associated with more significant postoperative pain, swelling, and reduced oral health‐related quality of life.^64^ In the present study, the analysis assessing the influence of clinician expertise, with graduate students serving as the reference group, demonstrated a statistically significant increase in horizontal bone gain when procedures were performed by expert surgeons. Previous reviews demonstrated that the number of similar cases treated by the same operator seemed to influence complication rates.^6^ However, when we contacted the authors of studies reporting ‘expertise level,’ they were unable to provide reliable counts of comparable prior surgeries, precluding quantitative analysis of surgeon expertise.

Vertical augmentation outcomes have also indicated increased bone gains with a pooled mean of 5.64 mm after 6 to 9 months follow‐up. However, as in the case of horizontal augmentation, the complication rate was high, making this procedure unpredictable. These findings generally align with other reports utilizing GBR approaches, showing slightly higher vertical bone gains than those previously reported with conventional techniques, which average around 4 mm^65^ and more closely align with GBR using customized CAD/CAM titanium meshes, which average around 5 mm.^21^ Subgroup analyses revealed that cases without a resorbable membrane tended to exhibit higher vertical gains compared to those with a membrane, which appears to contradict currently available direct evidence^66^ and may be more related to variations in the manuscripts included in this review. Finally, the type of defect influenced outcomes, with vertical and combined defects resulting in greater augmentation than purely horizontal defects, aligning with existing literature, irrespective of the technical complexity of vertical ridge augmentation in relation to horizontal augmentation.^6^, ^67^

The evaluation of volumetric bone gain demonstrated that using customized CAD/CAM titanium mesh produced a mean gain of 1307 mm^3^ overall, with subgroup analyses showing an increase to 1715 mm^3^ when a resorbable membrane was used. Our data indicated that vertical defects achieved more volumetric gains than horizontal defects. Also, the anatomical location (maxilla vs. mandible) affected volumetric outcomes, which is also supported by the literature demonstrating lower gains in maxillary sites.^65^ Interestingly, maxillary sites have also been shown to have a higher rate of complications following ARA.^68^ Currently, there remains uncertainty regarding the long‐term stability of achieved bone augmentation, particularly whether the initial bone gain is fully maintained or undergoes gradual resorption over time. This knowledge gap emphasizes the necessity for additional longitudinal studies to determine the clinical sustainability of regenerative outcomes.^8^

The pooled analysis of early complications revealed an overall early complication rate of 24.3%. These rates are higher than those previously reported in other systematic reviews for horizontal (17.8%)^69^ and vertical GBR (11%–16%^6^, ^70^). Notably, our analysis also observed considerable variability in late complications, particularly within different CAD/CAM device subgroups, echoing heterogeneous outcomes reported in previous investigations.^65^, ^71^ The rate of complications can also be associated with the technique used; membrane exposures were the most commonly reported adverse events during the healing phase when utilizing titanium meshes, regardless of whether conventional or customized titanium meshes were used.^72^ Although customized meshes demonstrated improved precision and defect adaptation, their use did not necessarily reduce the overall exposure rates compared to traditional meshes.^73^ Reported healing complication rates varied between 15% and 21.1%, with no statistically significant differences between groups using titanium‐reinforced membranes versus customized devices. Furthermore, while surgical complications were generally low, healing complications—especially major ones involving infection or significant membrane exposure—could compromise the regenerative process and occasionally necessitate additional interventions.^72^

Interestingly, a lower early complication rate of 21.4% was observed in studies using a resorbable membrane^18^, ^41^, ^44^ compared to 25.6% without a resorbable membrane.^18^, ^38^, ^45^ The use of a collagen membrane in combination with titanium mesh has been explored to reduce surgical complications and enhance horizontal bone gain in GBR; however, the results remain inconclusive. Recently, Urban and co‐workers reported that covering a perforated titanium‐reinforced PTFE mesh with a collagen membrane in vertical GBR did not significantly lower overall complication rates. It is worth mentioning that this and other studies by the same group using an additional collagen membrane noted minimal complications (3%) and an average vertical bone gain of 5.2 mm.^74^ In contrast, Borges et al. (2020) found that the adjunctive use of a collagen membrane did not improve bone quality or healing outcomes in an animal model.^75^ In a systematic review, Gu et al. (2022) demonstrated no significant difference in titanium mesh exposure rates when a collagen membrane was applied (23.9% with membranes vs. 20.2% without; *p* = 0.36), nor was there an observed improvement in horizontal bone gain.^76^ Likewise, Lim et al. (2015) showed that adding a collagen membrane failed to reduce mesh exposure or enhance mucosal healing and buccal bone preservation in a canine model.^77^

The majority of scaffolds assessed in this review had some sort of “pore system”.^18^, ^40^, ^45^, ^46^ These pores may support the revascularization of the grafted bone by enabling blood vessel infiltration from the surgical flap and enhancing interaction with the periosteum, which may aid in delivering progenitor cells to the site.^78^, ^79^, ^80^ However, the same perforations might compromise the scaffold's ability to serve as an effective barrier against undesirable cell migration, thereby potentially undermining the principle of cell occlusion central to GBR.^81^, ^82^ At the same time, despite enhancing vascular ingrowth, porous scaffolds may permit unwanted soft‐tissue infiltration, so a fully occlusive membrane still holds a lot of benefits in ARA.^83^

Data from recent systematic reviews show that surgeon experience and technique remain crucial determinants of success, as complication rates remain high regardless of digital customization, with differences in titanium mesh exposure rates between conventional and customized meshes (19.9% vs. 15.2%, *p* = 0.34).^76^ Similarly, Zhou et al. reported a 20% relative reduction in exposure rates when using customized meshes; however, the absolute exposure rate remained substantial at 31%.^84^ Hartmann and Seiler, likewise, documented a 25% exposure rate even when applying a digital workflow and using customized titanium meshes.^85^

These exposure rates align closely with those observed in the current study, suggesting that other clinical challenges (e.g., soft tissue phenotype, soft tissue management, and the common material (Titanium) cannot be merely mitigated by 3D printing and mesh customization.

Despite the clinically relevant dimensional gains that may be achieved with engineered scaffolds, several limitations temper the strength of our conclusions and point to avenues for future work. The heterogeneity among included studies, variations in study design, and differences in patient populations may restrict the generalizability of the findings. The review pooled different scaffold materials (titanium vs. PEEK vs. customized allogeneic bone blocks), with different scaffold “designs”. Only a single study was a randomized clinical trial that compared between a scaffold (CAD/CAM Ti‐ mesh) and a current gold‐standard (Ti‐ reinforced d‐ PTFE mesh).^46^ Remarkably, the reported evidence was imbalanced, with nine studies on titanium scaffolds versus only two on PEEK shells and one on 3D‐hydroxyapatite. This limits any comparative inference across materials. Pooled estimates are dominated by titanium‐mesh data and may reflect device‐ and operator‐specific effects rather than intrinsic material performance. Accordingly, our material subgroups are exploratory. We accordingly downgraded GRADE for indirectness and imprecision for non‐titanium scaffold modalities.

Most included studies relied on CAD/CAM–fabricated titanium meshes as barrier devices, underscoring that today's “engineered” scaffolds remain largely structural rather than truly bioactive matrices optimized for cell adhesion and migration; consequently, the elevated complication rates may reflect technique‐specific factors inherent to titanium mesh rather than intrinsic flaws in scaffold engineering.^86^, ^87^, ^88^ Follow‐up periods were generally short, leaving the long‐term stability of bone gains uncertain despite indirect inferences from graft material composition (e.g., autograft plus xenograft). Moreover, only a few studies incorporated patient‐reported outcome measures to capture functional and aesthetic satisfaction. Finally, the present contentious finding regarding the value of a resorbable membrane added to scaffolds, along with recent evidence supporting its usage,^66^ suggests that more RCTs are needed before a general recommendation can be made.

Future research should focus on larger, well‐controlled randomized trials to validate these observations and refine the understanding of how specific variables affect long‐term outcomes in bone augmentation. Future studies should prioritize outcomes such as the ability to place the implant, patient‐reported outcomes, and other implant clinical outcome assessments.^89^, ^90^ These should also prioritize the development and clinical testing of bioactive scaffold constructs,^91^, ^92^, ^93^ adopt standardized reporting frameworks, extend follow‐up to assess durability, and include both soft‐tissue and patient‐centered endpoints to fully characterize the clinical utility of engineered scaffolds, and conduct cost‐benefit analysis of using such scaffolds in ARA.

## 5 CONCLUSION

Within the limitations of this review, engineered scaffolds demonstrated effective gains in horizontal, vertical, and volumetric alveolar ridge augmentation. However, the procedure is technically challenging, with a high incidence of reported complications, both early and late. Key factors such as surgical experience, defect type (combined compared to pure vertical), and patient smoking status significantly impacted both bone gain and complication rates. These findings emphasize the need for advanced clinical skills, comprehensive training, and careful case selection when considering scaffold‐guided bone regeneration procedures.

## AUTHOR CONTRIBUTIONS

**Muhammad H. A. Saleh**: Methodology; investigation; writing—original draft; writing—review & editing; project administration. **Elena Calciolari**: Investigation; data curation; writing—original draft; writing—review & editing; supervision; project administration. **Hamoun Sabri**: Methodology; validation; formal analysis; writing—review & editing; visualization. **Ethan Ng**: Investigation; data curation; writing—original draft; writing—review & editing. **Sandra Stuhr**: Investigation; data curation; writing—original draft; writing—review & editing. **Nikolaos Donos**: Conceptualization; methodology; writing—review & editing; supervision; project administration. **Purnima Kumar**: Conceptualization; methodology; writing—review & editing; supervision; project administration.

## CONFLICT OF INTEREST STATEMENT

The authors report there are no conflicts of interest regarding the present work.

## FUNDING INFORMATION

No funding was received for this study.

## Supporting information



Supporting Information

Supporting Information

## Data Availability

The data that support the findings of this study have been included in the manuscript.
