# Supplementary material for: Efficacy of engineered scaffolds for horizontal and vertical ridge augmentation: A systematic review and meta‐analysis
Source: J Periodontol. 2026 Sep 14;97(Suppl 1):S33–51. doi: 10.1002/jper.70103 (PMC13575442; doi:10.1002/jper.70103)
Supplement: Supplementary file 1 [file JPER-97-S33-s001.docx]

**Supplementary Materials**

**Study title:** Efficacy of engineered scaffolds for lateral and vertical ridge augmentation: A systematic review and meta-analysis

# **Not for publication**

| **Section and Topic** | **Item #** | **Checklist item** | **Location where item is reported** |
| --- | --- | --- | --- |
| **TITLE** | | |  |
| Title | 1 | Identify the report as a systematic review. |  |
| **ABSTRACT** | | |  |
| Abstract | 2 | See the PRISMA 2020 for Abstracts checklist. |  |
| **INTRODUCTION** | | |  |
| Rationale | 3 | Describe the rationale for the review in the context of existing knowledge. |  |
| Objectives | 4 | Provide an explicit statement of the objective(s) or question(s) the review addresses. |  |
| **METHODS** | | |  |
| Eligibility criteria | 5 | Specify the inclusion and exclusion criteria for the review and how studies were grouped for the syntheses. |  |
| Information sources | 6 | Specify all databases, registers, websites, organisations, reference lists and other sources searched or consulted to identify studies. Specify the date when each source was last searched or consulted. |  |
| Search strategy | 7 | Present the full search strategies for all databases, registers and websites, including any filters and limits used. |  |
| Selection process | 8 | Specify the methods used to decide whether a study met the inclusion criteria of the review, including how many reviewers screened each record and each report retrieved, whether they worked independently, and if applicable, details of automation tools used in the process. |  |
| Data collection process | 9 | Specify the methods used to collect data from reports, including how many reviewers collected data from each report, whether they worked independently, any processes for obtaining or confirming data from study investigators, and if applicable, details of automation tools used in the process. |  |
| Data items | 10a | List and define all outcomes for which data were sought. Specify whether all results that were compatible with each outcome domain in each study were sought (e.g. for all measures, time points, analyses), and if not, the methods used to decide which results to collect. |  |
|  | 10b | List and define all other variables for which data were sought (e.g. participant and intervention characteristics, funding sources). Describe any assumptions made about any missing or unclear information. |  |
| Study risk of bias assessment | 11 | Specify the methods used to assess risk of bias in the included studies, including details of the tool(s) used, how many reviewers assessed each study and whether they worked independently, and if applicable, details of automation tools used in the process. |  |
| Effect measures | 12 | Specify for each outcome the effect measure(s) (e.g. risk ratio, mean difference) used in the synthesis or presentation of results. |  |
| Synthesis methods | 13a | Describe the processes used to decide which studies were eligible for each synthesis (e.g. tabulating the study intervention characteristics and comparing against the planned groups for each synthesis (item #5)). |  |
|  | 13b | Describe any methods required to prepare the data for presentation or synthesis, such as handling of missing summary statistics, or data conversions. |  |
|  | 13c | Describe any methods used to tabulate or visually display results of individual studies and syntheses. |  |
|  | 13d | Describe any methods used to synthesize results and provide a rationale for the choice(s). If meta-analysis was performed, describe the model(s), method(s) to identify the presence and extent of statistical heterogeneity, and software package(s) used. |  |
|  | 13e | Describe any methods used to explore possible causes of heterogeneity among study results (e.g. subgroup analysis, meta-regression). |  |
|  | 13f | Describe any sensitivity analyses conducted to assess robustness of the synthesized results. |  |
| Reporting bias assessment | 14 | Describe any methods used to assess risk of bias due to missing results in a synthesis (arising from reporting biases). |  |
| Certainty assessment | 15 | Describe any methods used to assess certainty (or confidence) in the body of evidence for an outcome. |  |
| **RESULTS** | | |  |
| Study selection | 16a | Describe the results of the search and selection process, from the number of records identified in the search to the number of studies included in the review, ideally using a flow diagram. |  |
|  | 16b | Cite studies that might appear to meet the inclusion criteria, but which were excluded, and explain why they were excluded. |  |
| Study characteristics | 17 | Cite each included study and present its characteristics. |  |
| Risk of bias in studies | 18 | Present assessments of risk of bias for each included study. |  |
| Results of individual studies | 19 | For all outcomes, present, for each study: (a) summary statistics for each group (where appropriate) and (b) an effect estimate and its precision (e.g. confidence/credible interval), ideally using structured tables or plots. |  |
| Results of syntheses | 20a | For each synthesis, briefly summarise the characteristics and risk of bias among contributing studies. |  |
|  | 20b | Present results of all statistical syntheses conducted. If meta-analysis was done, present for each the summary estimate and its precision (e.g. confidence/credible interval) and measures of statistical heterogeneity. If comparing groups, describe the direction of the effect. |  |
|  | 20c | Present results of all investigations of possible causes of heterogeneity among study results. |  |
|  | 20d | Present results of all sensitivity analyses conducted to assess the robustness of the synthesized results. |  |
| Reporting biases | 21 | Present assessments of risk of bias due to missing results (arising from reporting biases) for each synthesis assessed. |  |
| Certainty of evidence | 22 | Present assessments of certainty (or confidence) in the body of evidence for each outcome assessed. |  |
| **DISCUSSION** | | |  |
| Discussion | 23a | Provide a general interpretation of the results in the context of other evidence. |  |
|  | 23b | Discuss any limitations of the evidence included in the review. |  |
|  | 23c | Discuss any limitations of the review processes used. |  |
|  | 23d | Discuss implications of the results for practice, policy, and future research. |  |
| **OTHER INFORMATION** | | |  |
| Registration and protocol | 24a | Provide registration information for the review, including register name and registration number, or state that the review was not registered. |  |
|  | 24b | Indicate where the review protocol can be accessed, or state that a protocol was not prepared. |  |
|  | 24c | Describe and explain any amendments to information provided at registration or in the protocol. |  |
| Support | 25 | Describe sources of financial or non-financial support for the review, and the role of the funders or sponsors in the review. |  |
| Competing interests | 26 | Declare any competing interests of review authors. |  |
| Availability of data, code and other materials | 27 | Report which of the following are publicly available and where they can be found: template data collection forms; data extracted from included studies; data used for all analyses; analytic code; any other materials used in the review. |  |

# **Table S1. The search strategy**

**MEDLINE via OVID 1946 to November 21, 2024**

|  | MeSH | Free-text |
| --- | --- | --- |
| Population | Exp Dental Implants/  OR exp Dental Implantation  OR alveolar bone loss | dental implant* or (atroph* adj2 ridge) or (atroph* adj2 mandible) or (atroph* adj2 jaw) or bone dehiscence |
| Intervention/Comparison | alveolar ridge augmentation OR Exp bone regeneration OR exp guided tissue regeneration OR tissue scaffolds OR exp tissue engineering OR exp Printing, Three-Dimensional OR bioprinting | scaffold* OR “tissue engineering” OR ((3D and print*) and "bone regeneration") OR ridge augmentation OR (contour* AND augmentation) OR (regeneration AND (vertical OR horizontal OR lateral)) |

The search strategy was restricted to human studies by applying the double negation strategy suggested by the Cochrane handbook (i.e. combining the result with NOT “exp animals/ not humans.sh.”)

FIlter to exclude systematic reviews:

NOT (((systematic OR state-of-the-art OR scoping OR literature OR umbrella) ADJ (review* OR overview* OR assessment*)) OR "review* of reviews" OR meta-analy* OR metaanaly* OR ((systematic OR evidence) ADJ1 assess*) OR "research evidence" OR metasynthe* OR meta-synthe*).tw. OR systematic review/ OR "systematic review (topic)"/ OR meta analysis/ OR "meta analysis (topic)"/

Filter to exclude case reports and other non-relevant publications:

NOT (letter/ OR editorial/ OR news/ OR exp historical article/ OR anecdotes as topic/) OR (letter OR comment*.ti) OR case reports/

Filter to exclude guidelines:

NOT (clinical adj3 pathway).ti,ab,kw. or (clinical adj3 pathways).ti,ab,kw. or (practice adj3 parameter).ti,ab,kw. or (practice adj3 parameters).ti,ab,kw. or algorithms/ or care pathway.ti,ab,kw. or care pathways.ti,ab,kw. or clinical protocols/ or Consensus/ or Consensus Development Conference.pt. or Consensus Development Conference, NIH.pt. or Consensus Development Conferences as Topic/ or Consensus Development Conferences, NIH as Topic/or critical pathway/ or guidance.ti,ab. or guideline*.ti. or guidelines as topic/ or practice guidelines as topic/ or Health Planning Guidelines/ or practice guideline/

Filter to exclude cross-sectional studies:

Cross-Sectional Studies/ or Prevalence/ or (cross-sectional or prevalence or transversal).ti,ab,kw.

Filter to exclude other irrelevant studies

NOT (retrospective OR in vitro OR observational OR editorial OR Diagnostic Accuracy OR methodology OR economic OR sensitivity OR specificity OR orthopedic OR joint OR thoracic OR cardiac OR cancer)

Total hits 21st November 2024: 6029

**Embase**

|  | Emtree | Free-text |
| --- | --- | --- |
| Population | Exp tooth implant  OR exp alveolar bone loss | dental implant* or (atroph* NEAR/2 ridge) or (atroph* NEAR/2 mandible) or (atroph* NEAR/2 jaw) or bone dehiscence |
| Intervention/Comparison | Exp alveolar ridge augmentation OR exp bone regeneration OR exp tissue scaffold OR exp tissue engineering OR exp three dimensional bioprinting | scaffold* OR “tissue engineering” OR ((3D and print*) and "bone regeneration") OR ridge augmentation OR (contour* AND augmentation) OR (regeneration AND (vertical OR horizontal OR lateral)) |

Filter for human studies: NOT ([animals]/lim NOT [humans]/lim)

Filter to exclude systematic reviews:

NOT ((systematic OR state-of-the-art OR scoping OR literature OR umbrella) ADJ (review* OR overview* OR assessment*)) OR "review* of reviews" OR meta-analy* OR metaanaly* OR ((systematic OR evidence) NEAR/1 assess*) OR "research evidence" OR metasynthe* OR meta-synthe*.tw. OR “systematic review” OR "systematic review (topic)" OR meta analysis OR "meta analysis (topic)"

Total hits 21st November 2024: 4827

**CENTRAL**

|  | MeSH | Free-text |
| --- | --- | --- |
| Population | Exp Dental Implants/  OR exp Dental Implantation  OR alveolar bone loss | dental implant* or (atroph* NEAR/2 ridge) or (atroph* NEAR/2 mandible) or (atroph* NEAR/2 jaw) or bone dehiscence |
| Intervention/Comparison | alveolar ridge augmentation OR Exp bone regeneration OR exp guided tissue regeneration OR tissue scaffolds OR exp tissue engineering OR exp Printing, Three-Dimensional OR bioprinting | scaffold* OR “tissue engineering” OR ((3D and print*) and "bone regeneration") OR ridge augmentation OR (contour* AND augmentation) OR (regeneration AND (vertical OR horizontal OR lateral)) |

Total hits 21st November 2024: 1790

**SCOPUS**

“dental implant*” or (“atroph*” AND “ridge”) or (“atroph*” AND “mandible”) or (“atroph*” AND “jaw”) or “bone dehiscence”

AND

“scaffold*” OR “tissue engineering” OR ((“3D” and “print*”) and "bone regeneration") OR “ridge augmentation” OR (“contour*” AND “augmentation”) OR (“regeneration” AND (“vertical” OR “horizontal” OR “lateral”))

Filter for “article” and “journal” and “human”

Total hits 21st November 2024: 3890

# **Figure S1. PRISMA flow diagram.**

# ** These records did not fulfil the inclusion criteria during the Title/Abstract screening phase

# **Table S2. Excluded articles after full-text assessment, along with the reason for exclusion.**

| **N** | **Author and Year** | **Title** | **Reason for Exclusion** |
| --- | --- | --- | --- |
| **1** | **Kim et al 2024** | Customized three‑dimensional printed ceramic bone grafts for osseous defects: a prospective randomized study | No distinct primary outcome and included some advanced alveolar ridge preservation cases as guided bone regeneration. |
| **2** | **Mandelli et al 2021** | Customized-3D zirconia barriers for guided bone regeneration (GBR): clinical and histological findings from a proof-of-concept case series | No primary outcome |
| **3** | **Hartmann et al 2022** | Long-term results in three-dimensional, complex bone augmentation procedures with customized titanium meshes | No primary outcome |
| **4** | **Simion et al 2023** | Semi-occlusive CAD/CAM titanium mesh for guided bone regeneration: Preliminary clinical and histological results | No primary outcome |
| **5** | **Mangano et al 2014** | Maxillary Ridge Augmentation with Custom-Made CAD/CAM Scaffolds. A 1-Year Prospective Study on 10 Patients | No primary outcome |
| **6** | **Ghanaati et al 2019** | Biomaterial-based bone regeneration and soft tissue management of the individualized 3D-titanium mesh: An alternative concept to autologous transplantation and flap mobilization | No primary outcome |
| **7** | **Norton et al 2024** | Clinical and Histological Efficacy of Digitally Designed CAD CAM Allogenic Bone Blocks. A Prospective Cohort | No primary outcome |

# **Table S3. Accepted studies**

| **Reference** | **Author and Year** | **Title of publication included in the qualitative analysis (N=15)** | **N** | ***Quantitative Analysis (model included) (N=11)** |
| --- | --- | --- | --- | --- |
| **38** | **Cucchi et al 2020** | Clinical and volumetric outcomes after vertical ridge augmentation using computer-aided-design/computer-aided manufacturing (CAD/CAM) customized titanium meshes: a pilot study | **1** | Included/ Meta-analysis on complications |
| **18** | **Cucchi et al 2021** | Vertical and horizontal ridge augmentation using customized CAD/CAM titanium mesh with versus without resorbable membranes. A randomized clinical trial | **2** | Included (all models) |
| **44** | **Cucchi et al 2024** | A 3D micro-CT assessment of composition and structure of bone tissue after vertical and horizontal alveolar ridge augmentation using CAD/CAM-customized titanium mesh |  | Not included (Initial publication [Cucchi 2021] was included) |
| **41** | **Cucchi et al 2024** | Complication, vertical bone gain, volumetric changes after vertical ridge augmentation using customized reinforced PTFEmesh or Ti-mesh. A non-inferiority randomized clinical trial | **3** | Only Ti-Mesh arm was included/ Meta-analysis on complications |
| **43** | **Cucchi et al 2024** | Histological and histomorphometric analysis of bone tissue using customized titanium meshes with or without resorbable membranes: A randomized clinical trial |  | Not included (Initial publication [Cucchi 2021] was included) |
| **27** | **El-Morsy et al 2020** | Assessment of 3-dimensional bone augmentation of severely atrophied maxillary alveolar ridges using patient-specific polyether-ether ketone (PEEK) sheets | **4** | Included/meta-regression model |
| **36** | **De Santis et al 2022** | Custom Bone Regeneration (CBR): An Alternative Method of Bone Augmentation-A Case Series Study | **5** | Included/ All models |
| **37** | **Mekcha et al 2023** | Customized 3D printed nanohydroxyapatite bone block grafts for implant sites: A case series | **6** | Included/meta-regression model only |
| **39** | **Felice et al 2024** | A randomised controlled trial comparing the effectiveness of guided bone regeneration with polytetrafluoroethylene titanium-reinforced membranes, CAD/CAM semi-occlusive titanium meshes and CAD/CAM occlusive titanium foils inpartially atrophic arches | **7** | Only Ti-Mesh arm was included/ All models |
| **40** | **Giragosyan et al 2024** | Linear Bone Gain and Healing Complication Rate Comparative Outcomes Following Ridge Augmentation with Custom 3D Printed Titanium Mesh vs Ti-Reinforced dPTFE. A Randomized Clinical Trial | **8** | Only Ti-Mesh arm was included/ All models |
| **33** | **Lizio et al 2022** | Guided bone regeneration using titanium mesh to augment 3-dimensional alveolar defects prior to implant placement. A pilot study | **9** | Included/ All models |
| **34** | **Mohamed et al 2023** | Guided Bone Regeneration for Horizontal Maxillary Alveolar Ridge Augmentation Using Patient-Specific Solid Titanium Barriers: A Case Series | **10** | Included/Meta-regression model only |
| **42** | **Wang et al 2023** | Clinical and radiographic outcomes of customized allogeneic bone block versus autogenous bone block for ridge augmentation: 6 Month results of a randomized controlled clinical trial | **11** | Included/meta-regression model only |
| **35** | **Ciocca et al 2018** | Prosthetically CAD-CAM–Guided Bone Augmentation of atrophic Jaws Using Customized Titanium Mesh: Preliminary Results of an Open Prospective Study |  | Not included (the follow-up study [Lizio, 2022] was included) |
| **28** | **Mounir et al 2019** | Assessment of three-dimensional bone augmentation of severely atrophied maxillary alveolar ridges using pre-bent titanium mesh vs customized poly-ether-ether-ketone (PEEK)mesh: A randomized clinical trial |  | Not included |

***Note that the meta-analytic models to which each article was included are indicated in the fourth column.**

# **Table S4. Histological and Micro-CT parameters.**

| **Author and year** | **Histological parameters** |  | **MicroCT parameters** |  |
| --- | --- | --- | --- | --- |
|  | Interv 1 | Interv 2 | Interv 1 | Interv 2 |
| Mekcha et al 2023 | Histomorphometric analysis revealed that new bone formation, bone tissue, residual grafts, and connective tissue were 28.6 ± 1.88, 30.48 ± 4.81, 19.82 ± 4.07, and 20.81 ± 4.41%, respectively |  |  |  |
| Cucchi et al 2024a **(Cucchi et al 2021 Cohort)** | The bone tissue (B.Ar) was 34.3%, grafting material was (G.Ar) was 11.5%, and non-mineralized tissue (NMT.Ar) was 54.1%, and osteoid area (O.Ar) was 1.95 um2. | The bone tissue (B.Ar) was 35.3%, grafting material was (G.Ar) was 14.6%, and non-mineralized tissue (NMT.Ar) was 50%, and osteoid area (O.Ar) was 1.75 um2. |  |  |
| Cucchi et al 2024b (**Cucchi et al 2021 Cohort)** |  |  | 27.68% of bone tissue, 15.58% of biomaterial volume (MAT), and 25.24% of non-mineralized tissue volume (NMT) | 30.81% of bone tissue, 12.73% of biomaterial volume, and 25.42% non-­mineralized tissue volume (NMT) |

# **Table S5. RoB 2.0 for randomized controlled trials.**

Based on the RoB2 assessment, the risk of bias of each included RCT was rated as:

1. Low risk of bias (plausible bias unlikely to seriously alter the results) if all domains were at low risk of bias;

2. Unclear risk of bias (plausible bias that raises some doubt about the results) if ≥1 domains were at unclear risk of bias, but not to be at high risk of bias for any domain; or

3. High risk of bias (plausible bias that seriously weakens confidence in the results) if ≥1 domains were at high risk of bias, or the study is judged to have unclear risk of bias for multiple domains in a way that substantially lowers confidence in the result.

# **Table S6. ROBINS-I V2 for non-randomized studies.**

Based on the ROBINS-I V2 assessment, the overall judgment of risk of bias for each non-randomized study was rated as:

1. Low risk of bias, if overall there is a low risk of bias except for concerns about uncontrolled confounding in Domain 1 and low risk of bias in all other domains;
2. Moderate risk of bias, if at least one domain is at Moderate risk of bias, but no domains are at Serious risk of bias or Critical risk of bias;
3. Serious risk of bias, if at least one domain is at Serious risk of bias, but no domains are at Critical risk of bias, OR several domains are at Moderate, leading to an additive judgement of Serious risk of bias; or
4. Critical risk of bias, if at least one domain is at Critical risk of bias, OR several domains are at Serious risk of bias, leading to an additive judgement of Critical risk of bias.

# **Table S7. The certainty of evidence for the primary outcomes using adapted GRADE principles.**

| **Outcome** | **No. of Studies** | **Study Type** | **Risk of Bias** | **Inconsistency** | **Indirectness** | **Imprecision** | **Publication Bias** | **Certainty** |
| --- | --- | --- | --- | --- | --- | --- | --- | --- |
| Horizontal bone gain | 3 | RCTs & Case Series | Some concerns | Low (consistent results) | Low | Moderate | Unclear | ⬤⬤⬤◯ Moderate |
| Vertical bone gain | 5 | RCTs & Case Series | Some concerns | Moderate (membrane subgroup variation) | Low | Moderate | Unclear | ⬤⬤⬤◯ Moderate |
| Volumetric bone gain | 5 | RCTs & Case Series | Some concerns | Serious (high interstudy range) | Low | Serious (wide CI, small N) | Unclear | ◯⬤◯◯ Low |
| Early complications | 6 | RCTs & Case Series | Serious | Serious (range 0–41%) | Low | Serious | Unclear | ◯⬤◯◯ Low |
| Late complications | 3 | RCTs & Case Series | Serious | Serious | Low | Serious (wide CI, low N) | Unclear | ◯⬤◯◯ Low |
