# Supplementary material for: Efficacy of engineered scaffolds for horizontal and vertical ridge augmentation: A systematic review and meta‐analysis
Source: J Periodontol. 2026 Sep 14;97(Suppl 1):S33–51. doi: 10.1002/jper.70103 (PMC13575442; doi:10.1002/jper.70103)
Supplement: Supplementary file 2 [file JPER-97-S33-s002.docx]

| **STUDY CHARACTERISTICS** | | | | | | | **POPULATION CHARACTERISTICS** | | | | | | | | | | **Defect characteristics** | | | **TYPES OF INTERVENTIONS AND COMPARISONS** | | | | | | **Surgery Specifics** | |  |  |
| --- | --- | --- | --- | --- | --- | --- | --- | --- | --- | --- | --- | --- | --- | --- | --- | --- | --- | --- | --- | --- | --- | --- | --- | --- | --- | --- | --- | --- | --- |
|  |  |  |  |  |  |  |  |  |  |  |  |  |  |  |  |  |  |  |  |  |  |  |  |  |  |  |  |  | |
|  |  |  |  |  |  |  |  |  |  |  |  |  |  |  |  |  |  |  |  |  |  |  |  |  |  |  |  |  | |
| **Author and year** | **Study Design** | **Country Study conducted** | **Setting (Hospital/University/private practice)** | **Ethics approval reported (YES/NO/NI)** | **Funding** | **Study fulfils criteria for FQ1, FQ2, FQ3, FQ4?** | **Demographics** | | | | | | | **Periodontal status** | | | **simultaneous/ 2-stage regeneration** | **Bone defect distribution (maxilla/mandible)** | **Bone defect characteristics (horizontal, vertical, combined); number of teeth involved** | **INTERVENTION 1** | | | **INTERVENTION 2** | | | **Surgeon(s) experience** | **Study follow-up** |  |  |
|  |  |  |  |  |  |  | **Patients Enrolled (n)** | **Age in Years (mean ± sd; range)** | **Male/female** | **Ethnicity** | **Smoking status (smokers/nonsmokers/ex-smokers)** | **Bruxism (Yes/No; number of bruxers)** | **Drop-outs/losses at follow-up (n)** | **History of periodontitis** | **Periodontal status at the time of the study** | **FMPS/FMBS at baseline** |  |  |  | **Type of biomaterial(s) used** | **How was the scaffold stabilized (e.g. pins, sutures?)** | **Use of post-op provisionals** | **Type of biomaterial(s) used** | **How was the scaffold stabilized (e.g. pins, sutures?)** | **Use of post-op provisionals** |  |  |  |  |
| **Lizio 2022 (including the population of Ciocca et al 2018)** | Prospective case series | Italy | Hospital | YES | no financial support | FQ2 and FQ4 | 17 | 55.9±13.7 | 5/12 | NR | smokers of <10 cigarettes included (unclear number) | NR | 0 | NR | NR | NR | 2-stage | 8 in maxilla (1 posterior associated with sinus lift and 7 anterior) and 11 in posterior mandible | Combined | customized titanium mesh scaffold with milled autologous bone block mixed with deproteinized bovine bone matrixin a 60/40 ratio | Two or three titanium screws | NR |  |  |  | different expertise and experience | 6 months |  |  |
| **Mohamed et al. 2023** | Prospective case series | Egypt | University | YES | self-funded | FQ2 | 8 | 28.3 (18 to 40) | 4/4 | NR | All NS | NR | 0 | NR | NR | NR | 2-stage | atrophic anterior maxillary ridges | horizontal | an equal mix of autogenous cancellous particulates and xenogenic bone incorporated into a solid nonperforated concealing CAD/CAM fixating grade IV titanium barrier. | four to six mini-screws | NR |  |  |  | Post-Graduate Clinical PhD student* | 4 and 10 months |  |  |
| **De Santis et al 2022** | Prospective case series | Italy | Hospital | YES | No external funding | FQ2 and FQ4 | 9 | 53.1 ± 6.8 | 6F, 3M | NR | NR | NR | 0 | NR | NR | NR | 2-stage | 4 posterior mandible, 4 anterior maxilla, one posterior maxilla | Combined horizontal and vertical defects | CAD-CAM customised titanium mesh filled with autologous bone and deproteinised bovine bone in a 50:50 ratio | Mini titanium screws | NR | N/A |  |  | NR | 9 months |  |  |
| **Mekcha et al 2023** | Prospective case series | Thailand | University | YES | Funded by the National Research Council of Thailand. No external funding | FQ2 | 12 | 53.5 +/- 9.3 | 6F, 6M | NR | NR | NR | 0 | NR | Controlled | NR | 2-stage | 9 anterior maxilla, 3 posterior mandible | Horizontal | 3D hydroxyapatitite block, particulate bovine bone graft, collagen membrane, PRF membrane | No fixation screw needed because it completely fit the defect without movement | 8/12 received a provisional | N/A |  |  | Third year PG student in the master of implantology degree, who was closely supervised by an experienced specialist | 6 months |  |  |
| **Felice et al 2024** | RCT, single-centre | Italy | University | YES | NR (but no COI declared) | FQ2 and FQ4 | 27 | 56.37 +/- 10.34 | 15F, 12M | NR | 14.8% smokers (not heavy smokers) | NR | 0 | NR | NR | NR | 2-stage | Posterior mandible 13 (48.2%), anterior mandible 4 (14.8%), posterior maxilla 8 (30%), anterior maxilla 2 (7.4%) | Combined horizontal and vertical | 80:20 mixture of particulate autogenous bone chips and porcine-drived xenograft, Titanium-reinforced PTFE | osteosynthesis screws | NR | 80:20 mixture of particulate autogenous bone chips and porcine-drived xenograft. Titanium-reinforced PTFE or customised CAD/CAM occlusive/semiocclusive titanium devices. The titanium devices were covered with a resorbable membrane | Osteosynthesis screws (only required on the buccal) | NR | All surgical procedures were performed by three expert surgeons | 8 months |  |  |
| **Giragosyan et al 2024** | RCT, single-centre | Bulgaria | University | YES | NR | FQ2 and FQ4 | 40 | NR | NR | NR | NR | NR | 0 | NR | NR | NR | 2-stage | NR | combined horizontal and vertical | Xenograft and autogenous bone in a 50:50 ratio Ti-reinforced dPTFE | Titanium screws | NR | Xenograft and autogenous bone in a 50:50 ratio Customised 3D printed titanium mes | Titanium screws | NR | NR | 6 months |  |  |
| **Mounir et al 2019** | RCT, single-centre | Egypt | University | YES | NR | FQ2 and 4 | 16 (8 Int 1, 8 Int 2) | 38 for Int 1, 39 for Int 2 | Int 1: 6/2; Int 2: 4/4 | NR | NR | NR | 0 | NR | NR | NR | 2-stage | 100% maxilla | Combined horizontal and vertical | prebent Ti mesh with 50:50 mix of particulate autogenous (iliac crest) and xenogenic bone graft | micro titanium screws | NR | prebent Ti mesh with 50:50 mix of particulate autogenous (iliac crest) and xenogenic bone graft | micro titanium screws | NR | Post-Graduate Clinical PhD student* | 6 months |  |  |
| **Wang et al 2023** | RCT, single-centre | China | University | YES | YES | FQ2, FQ4 | 24 (12 Int 1, 12 Int 2) | 38.67 +/- 15.20 for Int 1, 29.83 +/- 8.61 for Int 2 | Int 1: 6/6 ; Int 2: 4/8 | NR | Int 1: 2/12 ; Int 2: 1/12 | NR | 0 | NR | probing pocket depths had to be <4 mm with BOP < 10% | FMPS <25% ; FMBS <10% | 2-stage | Did not specify maxilla vs mandibular, but specified molar vs non molar vs incisor | Combined; ;no more than 4 missing teeth | Customized allogeneic bone block (CABB), allograft bone particulate, collagen membrane | titanium pins (8mm long, 1.5mm diameter) | NR | autogenous block graft from ramus, allograft bone particulate, collagen membrane | titanium pins | NR | All surgeries completed by one experienced surgeon* | 6 months |  |  |
| **Cucchi et al 2020** | Prospective case series | Italy | University | YES | Self-Funded | FQ4 | 10 | 52 | 5M, 5F | NR | 2 | NR | 0 | 5 | NR | NR | 2-stage | 2 ant maxilla; 3 post maxilla; 1 aterior mandible, 4 post mandible | Vertical defects. partial edentulism, 1-4 implants | Customized CAD/CAM titanium mesh + 50% harvested autologous bone with 50% of xenograft | titanium mini-screws | NR | N/A | N/A | N/A | All surgeries completed by one experienced surgeon* | 6-8 months |  |  |
| **Cucchi et al 2021 & 2024a, b** | Mono centric, non-­ inferiority, RCT | Italy | University | YES | NR | FQ2 and FQ4 | 30 (15 Int 1, 15 Int 2) | NR | Intervention 1: 7/15; Intervention 2: 8/15 | NR | NR | NR | 0 | NR | NR | NR | 2-stage | 7 (23.3%) in the anterior region (1 mandible, 6 maxilla), 23 (76.7%) in the posterior region (15 mandible, 8 maxilla) | Combined horizontal and vertical defects (25 verical and 5 horizontal); partial edentulism | customised CAD-CAD titanium mesh filled with 50:50 autogenous bone and xenograft | osteosynthesis screws | no removable partial dentures for the entire healing period | customised CAD-CAD titanium mesh overfilled with 50:50 autogenous bone and xenograft + cross-linked collagen membrane | osteosynthesis screws | no removable partial dentures for the entire healing period | All surgeries completed by one experienced surgeon* | 6 months |  |  |
| **Cucchi et al 2024 (Ti-Mesh group)** | Mono centric, non-­inferiority, double-­blind, parallel-­group RCT. | Italy | University* | YES | No funding received | FQ4 | 25 | 57 | (5 males, 20 females) | NR | 1 (4%) | NR | 1 | 8 (32%) | Only treated periodontal patients were included | NR | 2-stage | 12 maxilla, 12 mandible | Purely vertical 11 (45.8%), 13 combined vertical and horizontal; all partial edentulism | A 50:50 mixture of autogenous bone (harvested using the bone scraper technique and deproteinized bovine bone matrix (DBBM) + 3-­mL of peripheral venous blood were placed in Customized CAD/CAM titanium mesh | Osteosynthesis screws | During the entire healing period, removable partial dentures were not used to prevent interference with surgical site healing. | N/A | N/A | N/A | Experienced in VRA | A CBCT scan was done at 6 months for mandibular de- fects and at 9 months for maxillary defects |  |  |
| **El-Morsy et al. 2020** | Prospective case series | Egypt | University* | YES | NR | FQ2 and FQ4 | 14 | 29 | (5 males, 9 females) | NR | NR | NR | 0 | NR | NR | NR | 2-stage | 14 mandible | combined horizontal and vertical | A 50:50 mixture of autogenous bone (harvested using ACM from a second site (chin or retromolar) + deproteinized bovine bone matrix (DBBM; and were placed in Customized CAD/CAM in-house printed PEEK shells | Micro-titanium screws | NR | N/A | N/A | N/A | Post-Graduate Clinical PhD student* | 6 months |  |  |

**Table 1**.  Characteristics of Studies Included in the Systematic Review and Meta-Analysis. ***** Not reported in the publication but information was gained by direct contact with the study authors; NR= Not reported; NA= non-applicable.
